# Wafer-Level Vacuum Packaging of Smart Sensors

**DOI:** 10.3390/s16111819

**Published:** 2016-10-31

**Authors:** Allan Hilton, Dorota S. Temple

**Affiliations:** Electronics and Applied Physics Division, RTI International, Research Triangle Park, NC 27709, USA; temple@rti.org

**Keywords:** wafer-level vacuum packaging, hermetic packaging, wafer-to-wafer bonding, smart sensors, microelectromechanical (MEMS), eutectic bonding, solid-liquid interdiffusion (SLID) bonding, metal-metal bonding, thin-film getter, AuSn, CuSn, AuSi, AlGe, AuIn

## Abstract

The reach and impact of the Internet of Things will depend on the availability of low-cost, smart sensors—“low cost” for ubiquitous presence, and “smart” for connectivity and autonomy. By using wafer-level processes not only for the smart sensor fabrication and integration, but also for packaging, we can further greatly reduce the cost of sensor components and systems as well as further decrease their size and weight. This paper reviews the state-of-the-art in the wafer-level vacuum packaging technology of smart sensors. We describe the processes needed to create the wafer-scale vacuum microchambers, focusing on approaches that involve metal seals and that are compatible with the thermal budget of complementary metal-oxide semiconductor (CMOS) integrated circuits. We review choices of seal materials and structures that are available to a device designer, and present techniques used for the fabrication of metal seals on device and window wafers. We also analyze the deposition and activation of thin film getters needed to maintain vacuum in the ultra-small chambers, and the wafer-to-wafer bonding processes that form the hermetic seal. We discuss inherent trade-offs and challenges of each seal material set and the corresponding bonding processes. Finally, we identify areas for further research that could help broaden implementations of the wafer-level vacuum packaging technology.

## 1. Introduction

Miniaturized semiconductor sensors are at the heart of the next wave of interconnectivity—the transition from the “Internet of People” to the Internet of Things (IoT). To realize the full potential of IoT, sensor fabrication methods must continue to reduce the size, weight, power, and cost (SWaP-C) of the sensor component and system. The same trend needs to apply to sensor packaging, which currently accounts for as much as 80% of the overall cost and form factor. To make IoT a reality, the industry needs to adopt packaging methods that reduce SWaP-C.

Many of semiconductor sensors, especially those in the category of microelectromechanical systems (MEMS), require a vacuum environment to achieve the desired sensitivity. In this group are inertial sensors, thermal imagers, pressure sensors, and resonance devices. The vacuum environment is typically established soon after the sensor fabrication to protect the sensor during downstream assembly. To reduce SWaP-C, vacuum packaging has shifted from serial, die-level approaches to massively parallel, wafer-level vacuum packaging (WLVP). In a WLVP process, a wafer containing a semiconductor sensor die (i.e., device wafer) is bonded under vacuum to a passive lid wafer or to another device wafer containing part of the sensor architecture.

**Figure 1:**
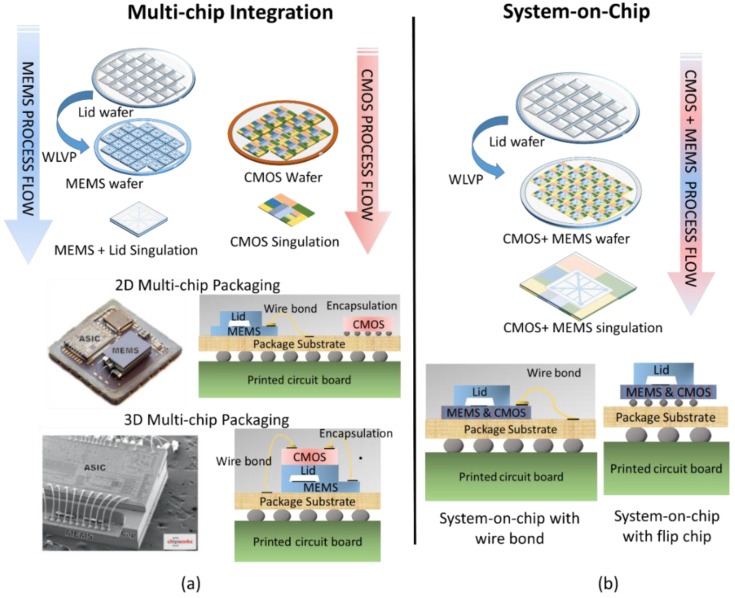
(**a**) Examples of multi-chip architecture in which vacuum-packaged microelectromechanical systems (MEMS) sensors are connected to complementary metal-oxide semiconductor (CMOS) integrated circuits (ICs) through the package substrate. Includes both two-dimensional (2D) and three-dimensional (3D) integration examples; (**b**) Example of system-on-chip (SoC) architecture in which wafer-level vacuum packaging (WLVP) is used to place MEMS and CMOS in the same package. Photograph of 2D multi-chip package reprinted with permission from Colibrys Ltd, (Yverdon-les Bains, Switzerland). Scanning electron microscope (SEM) image of STMicroelectronics three-axis MEMS accelerometer reprinted with permission from Chipworks [1].

Smart sensors form when sensor elements are closely integrated with silicon (Si) integrated circuits (ICs). These readout ICs (ROICs) provide device bias, signal amplification, and other signal processing functions. Originally, WLVPs included only discrete sensor devices, and smart sensors were realized by connecting discrete MEMS chips to ROIC chips through the package or board substrate. This approach, depicted in Figure 1a, is commonly referred to as multi-chip integration and has many different embodiments in both two-dimensional (2D) and three-dimensional (3D) formats. Figure 1b shows the WLVP process flow for the case where the complementary metal-oxide semiconductor (CMOS) IC and sensor elements are interconnected directly, without the use of routing layers in the package or board, in a construct referred to as a system-on-chip (SoC).

The SoC approach is required for large-format pixelated devices such as micro-mirrors and imaging arrays. By monolithically integrating sensor arrays with CMOS ICs, one can achieve a pixel-level interconnect density. When compared to the discrete multi-chip packaging approach, SoC is typically more complex but leads to reduced parasitics, smaller footprints, higher interconnect densities, and lower package costs. Although SoC is still primarily implemented in pixelated devices, some mature, high-volume microphones, pressure sensors, and inertial sensors are shifting from multi-chip to SoC embodiments to reduce SWaP-C [2].

This fusion of MEMS and CMOS ICs at the chip level creates new challenges for the WLVP process. These challenges result from the requirement of thermal, chemical, and mechanical compatibility of the WLVP process with not only the sensing element but also the CMOS ICs. Several WLVP approaches have been developed to address these challenges. In this paper, we review progress in the development of WLVP, discussing techniques that have been reported for the deposition of seals, wafer-to-wafer bonding, and getter deposition and activation, and we compare the different approaches in the context of device requirements. Although a number of comprehensive papers have been published on the related topics of CMOS and MEMS integration [2,3,4,5,6], wafer-level packaging (WLP) of MEMS [5,6,7,8,9,10,11,12], and WLVP of MEMS [10,13,14,15,16], no review has addressed the WLVP of SoC smart sensors. In the cases where topical overlap exists, more recent and/or more detailed information is provided.

In the first section of the paper, we describe the generic WLVP process. In the second section, we discuss wafer-to-wafer bonding approaches, seal material sets, and deposition methods used for seal fabrication. In the third section, we address the deposition of getter material and corresponding activation methods. Our review ends with comments regarding future directions of the WLVP technology in smart sensor implementations.

## 2. Introduction to WLVP Process

**Figure 2:**
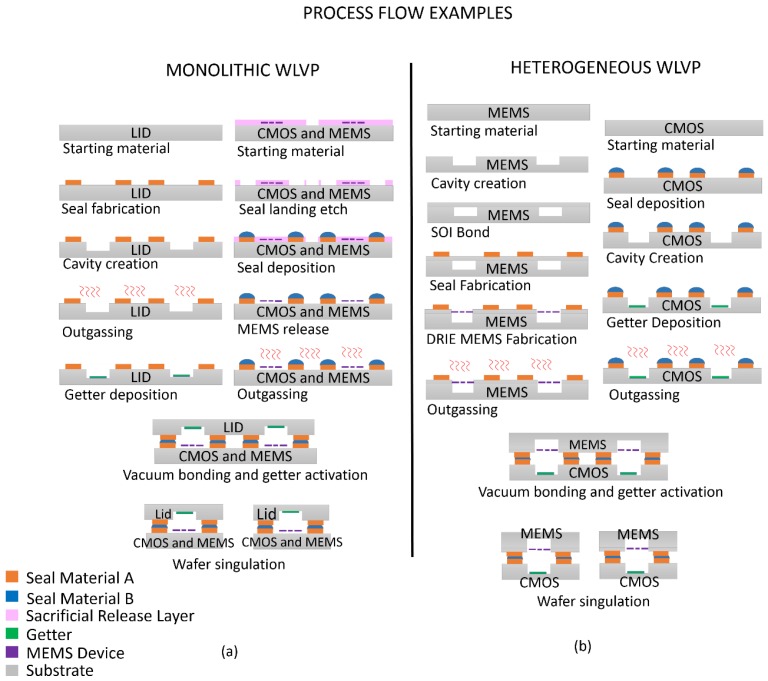
Examples of wafer-level vacuum packaging (WLVP) process flows. (**a**) Monolithic example in which complementary metal-oxide semiconductor (CMOS) and microelectromechanical systems (MEMS) are capped with a passive lid wafer; (**b**) Heterogeneous example in which CMOS and MEMS are on separate wafers, which are bonded to form a WLVP.

SoC smart sensor fabrication begins with the CMOS and MEMS fabrication processes, which can be integrated monolithically or heterogeneously, as illustrated in Figure 2. In the monolithic approach (Figure 2a), MEMS and CMOS devices are fabricated in a serial or parallel fashion on the same substrate. The device wafer is bonded to a lid or cap wafer to create sealed packages. In the heterogeneous approach (Figure 2b), the CMOS and MEMS devices are created on separate wafers. Bonding of the wafers creates the vacuum package, or the vacuum package can be created after the integration of MEMS and CMOS. More information on various CMOS and MEMS SoC integration schemes can be found in recently published reviews [2,6]. Regardless of the integration approach, the device designers and fabricators have to overcome many of the same WLVP integration challenges.

The WLVP process typically incorporates the fabrication of cavities in one or both of the wafers; cavities provide an unoccupied space in which the MEMS device operates. They also increase the internal package volume, making it easier to achieve the desired ambient pressure. For reasons to be discussed in detail below, metal-based seals—the primary focus of this paper—are typically used in WLVP. The order in which seals and cavities are created is highly dependent on the integration approach. If the seal material is to be deposited after cavity creation, the resulting topography requires that the metal layer is patterned using shadow masks, conformal resist deposition techniques such as spray coating, or dry-film resist lamination. Traditional, less complex, spin-coated resists can be used if the seal metal is deposited prior to cavity creation. In this case, however, the seal metal must be compatible with the cavity etching process. Metal deposition methods discussed in Section 3 will impact these integration decisions.

As shown in Figure 2, thin film getters are typically incorporated into the package to reach and maintain the desired pressure. Once activated, these getters absorb non-noble atmospheric and other gases that diffuse out of electronic devices. The getter activation typically occurs at temperatures of at least 300 °C–400 °C [17]. To prevent premature activation, wafers receiving the getters should be outgassed prior to the getter deposition.

The release of MEMS devices is the last step before bonding. The release usually involves dry or wet etching of a sacrificial layer used in device fabrication. If getter is deposited on the MEMS device wafer, the release process must not adversely impact getter functionality. In addition, the release process needs to be compatible with the exposed seal materials. Any sealing surface that is sensitive to the release process should be incorporated on the non-MEMS wafer, if possible.

Once the deposition and patterning of the seal metal, the deposition of a getter, and the MEMS release are complete, the wafers are ready for bonding. Bonding should be carried out in a vacuum bonder capable of heating the wafers and applying mechanical force. The getter can either be activated before, during, or after bonding, depending on the bonding equipment and wafer temperature limitations. These options will be discussed in more detail in later sections.

## 3. Bonding Approaches

Many of the traditional, discrete MEMS bonding approaches (e.g., direct, anodic, and glass frit bonding) are incompatible with the WLVP of smart sensors.

Direct bonding, also known as fusion bonding, relies on the formation of covalent bonds between Si and silicon dioxide (SiO_2_) substrates. This type of bonding is typically performed at temperatures greater than 700 °C [18]. Through the use of surface treatments (e.g., wet cleans and exposure to ionized plasma), CMOS-compatible direct bonding has been demonstrated below 400 °C [19,20,21]. Direct bonding relies on weak physical forces that vanish at distances of just a few nanometers (nm), so the surfaces to be bonded have to be very smooth, with roughness values below 0.3–0.5 nm [22,23,24]. The sensitivity to the surface roughness, high bonding temperatures, and relatively high permeation rates of gases in SiO_2_ limit the applicability of this bonding approach to the WLVP of smart sensors.

Anodic bonding is a commonly used process to bond glass lid wafers to Si-based MEMS devices. The glass is a borosilicate glass containing a high concentration of alkali ions. When an electric field is created between Si and the glass, the ions migrate to the cathode, leaving behind a fixed negative charge [25,26]. The resulting electrostatic force brings the wafer surfaces into intimate contact. The wafers are then heated to 300 °C–450 °C, and the wafer surfaces react, forming strong Si-O-Si bonds [27]. Due to the high voltages of 1000 V–2000 V applied during the bonding process, as well as discharge effects, the risk of sodium (Na+) contamination, and stresses resulting from high temperature bonding of dissimilar substrates, it is very difficult to create a CMOS-compatible, anodic bonding process [28]. Furthermore, this approach applies to a limited set of substrate materials [26].

Another traditional approach commonly used for MEMS packaging gets its name from the intermediate bonding layer made of glass frit [29]. The frit is printed through a stencil on one of the substrates prior to bonding. Due to limited control over the frit deposition process and the flow of frit during bonding, one needs seal exclusion areas of hundreds of micrometers (μm) in width [30]. These large areas take up valuable wafer real estate, reducing the number of die per wafer and increasing overall device fabrication costs. In addition, significant outgassing from glass frit occurs during the bonding process, making it a poor candidate for devices that require low package pressures.

Table 1 summarizes challenges associated with the bonding processes described above, and compares them with metal-based bonding approaches that have been developed to address shortcomings of traditional bonding. The metal-based approaches, described in detail below, leverage mature deposition and patterning techniques developed for ICs and MEMS. These mature fabrication techniques combined with the relatively low permeability of gases in metals result in hermetic sealing processes with as little as a few micrometers of the seal material. These small seals reduce chip real-estate required for bonding and therefore reduce the cost of the component. The fabrication of the seals prior to bonding allows for outgassing of the seal material, reducing the package pressure. Metal-based bonding approaches can be executed at temperatures compatible with CMOS circuits and integrated with various substrate materials and topographies, making them ideal for smart sensor packaging.

Metal-based hermetic bonding approaches fall largely into three categories: eutectic, solid-liquid interdiffusion (SLID), and metal-to-metal. Table 2 lists the primary advantages and disadvantages of each approach, and Section 3.1, Section 3.2 and Section 3.3 discuss each one in detail.

### 3.1. Eutectic Bonding

This bonding approach is defined by the presence of an intermediate bonding layer of a metallic alloy at eutectic composition. During bonding, once the eutectic melting temperature is exceeded, the alloy undergoes a phase transformation from solid to liquid. Once the liquid layer forms, the intermediate bonding layer collapses, accommodating surface roughness, particles, and other sources of non-uniformity between the two wafers. The liquid flows to all wettable surfaces, creating a continuous sealing layer that solidifies during cooling.

Table 3 outlines the most common eutectic alloys used for hermetic and/or vacuum packaging of IC, MEMS, and smart sensing devices, and their corresponding eutectic temperatures. For WLVP of smart sensors, alloys with eutectic temperatures at or below 400 °C are desired. This allows the bonding temperatures, which are typically 20 °C–50 °C above the eutectic temperature, to remain below the CMOS thermal budget limitations of approximately 450 °C for a period of minutes. For devices that require low package pressures for operation, it is also advantageous to outgas the wafers above the bonding temperature prior to bonding, which further limits the bonding temperature.

#### 3.1.1. Gold-Tin (Au-Sn) Eutectic Bonding

Due to a number of inherent advantages, Au_0.8_Sn_0.2_ eutectic bonding has been heavily researched for WLVP packaging and demonstrated using surrogate proxy wafers [31,32,33,34,35,36], RF MEMS switches [37,38], MEMS resonators [39], and infrared imaging smart sensors [40]. In this approach, Au_0.8_Sn_0.2_ is deposited on either the lid or device wafer, and a wettable layer, typically Au, is deposited on the other wafer. Since Au_0.8_Sn_0.2_ bonding layers do not oxidize, one does not need to clean the surface with flux or other chemicals that might be incompatible with released MEMS devices. The inertness of Au also makes the Au_0.8_Sn_0.2_ eutectic an ideal solution for devices used in corrosive environments (e.g., in medical applications) [41]. In addition, Au_0.8_Sn_0.2_ is a relatively hard, high-yield-strength solder, resistant to thermomechanical fatigue such as creep.

The primary challenges associated with Au_0.8_Sn_0.2_ eutectic bonding are rooted in the steep liquidus lines, particularly on the Au side of the eutectic composition of interest. At 81% and 75% Au by weight, the melting temperature is equal to 340 °C and 333 °C, respectively [34]. To realize the melting temperature of 280 °C, the alloy composition must be precisely controlled, which creates process control challenges during deposition and bonding.

**Figure 3:**
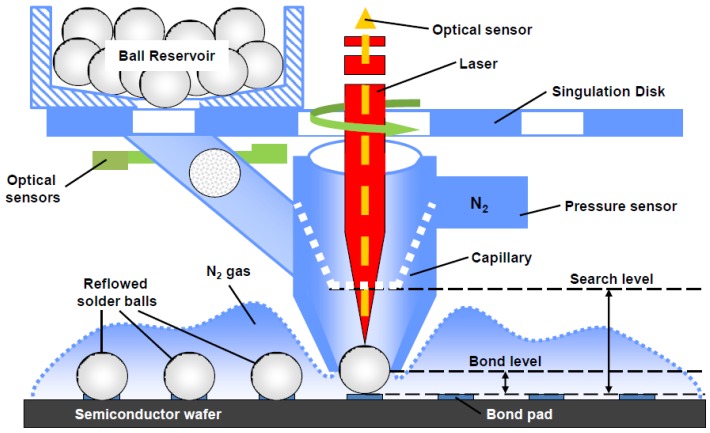
Diagram depicting the solder jetting process [43]. Reprinted with permission from PacTech.

Table 4 lists techniques that are used for Au_0.8_Sn_0.2_ deposition. These include solder ball jetting, alloy electroplating, and multilayer Au/Sn structure deposition. The ball jetting process involves the use of a preform at the desired alloy composition. In this approach, illustrated in Figure 3, bulk Au_0.8_Sn_0.2_ solid is melted, and solder spheres are formed and ejected on the under-seal metal (USM) layer at a rate of several balls per second [42]. During bonding, which happens at temperatures greater than the eutectic temperature, the spheres melt and wet the USM layer, forming a continuous, hermetic seal. The thickness of the bond is determined by a combination of the ball diameter, bonding force, pad geometry, and wetting characteristics of the USM. These variables must be carefully balanced to ensure uniform seal formation across each die and wafer, without excess squeeze-out of the material that may impact active device regions. The challenges associated with controlling the seal geometry make this bonding approach unattractive for solutions that require narrow seal widths. This approach may also be cost-prohibitive for high-volume markets due the serial nature of the ball placement and the relatively high material cost.

Electroplating of the Au_0.8_Sn_0.2_ eutectic alloy through a resist pattern is an attractive deposition method due to the composition and geometric control that it affords. However, the approach has significant challenges. Due to inherent non-uniformities of the electric field in any plating cell, electroplating deposition rates vary across the wafer. These field variations, in combination with relatively large differences in electrode potentials between Au and Sn, make it difficult to control the relative amounts of the metals in the deposit. Although a number of groups have worked on developing electroplating solutions [44,45,46,47,48], information on compositional uniformity is very limited. The limited data suggest that the composition uniformity range is as much as 6.6% from wafer to wafer [47]. This range results in melting temperature differences as high as 50 °C, making it difficult to create a reproducible bonding process.

**Figure 4:**
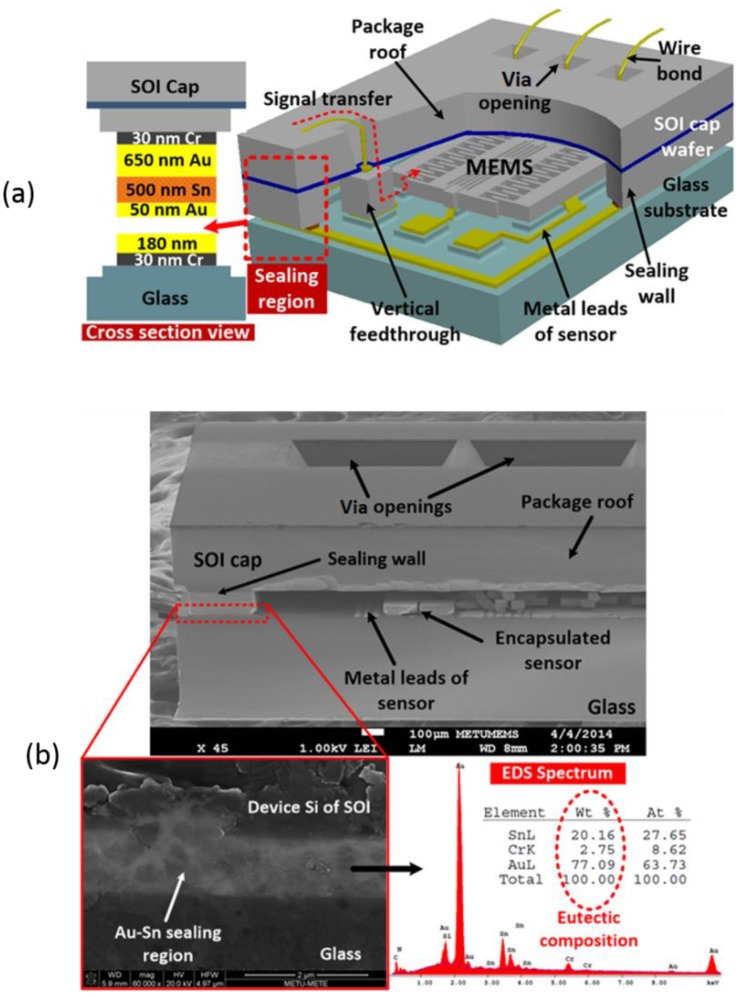
Alternating layers of evaporated Au and Sn are used to form the Au_0.8_Sn_0.2_ eutectic bond for the wafer-level packaging (WLVP) of a microelectromechanical (MEMS) resonator [35]. (**a**) Cartoon depicting the embodiment; (**b**) Scanning electron microscope (SEM) image of the completed structure including the Au_0.8_Sn_0.2_ eutectic bond. Reprinted from ref. [35] with permission from the Institute of Electrical and Electronics Engineers (IEEE).

The third approach listed in Table 4 involves the deposition of alternating layers of pure Au and Sn [49,50]. Once the layers are deposited, the stack is annealed to create the Au_0.8_Sn_0.2_ eutectic material. This deposition approach has been demonstrated for both the IC interconnect [41,51,52] and hermetic MEMS packaging [31,32,33,37,38]. Figure 4 depicts an implementation of this approach in the fabrication of a WLVP for an RF resonator device with vertical feedthroughs [35].

Deposition of the alternate layers is most commonly done by evaporation or electroplating. If Au is deposited on top of the layered structure, using the evaporation technique reduces the possibility of Sn oxide formation, which can result in voids in the subsequent bond. The evaporation approach also provides more control over the thicknesses of the alternating layers and therefore the composition of the alloy. When bonding layers thicker than 1 to 2 μm are needed, electroplating becomes more cost-effective.

Once the alternating layers are deposited, either by evaporation or electroplating, annealing is used to promote the homogeneous mixing and formation of Au_0.8_Sn_0.2_. Annealing has been demonstrated at different dwell temperatures, at different ramp rates, and in different ambients [41,52]. The anneal process can be carried out as a separate step or as part of the bonding procedure. During bonding, the Au_0.8_Sn_0.2_ material is brought into contact with the wettable USM layer on the mating wafer and heated to 10 °C–50 °C above the melting temperature. In general, the USM acts as a barrier to prevent Sn and Au from diffusing into Si. The USM also acts as an adhesion and/or wetting layer. When electroplating is used for the Au-Sn deposition, a seed layer is also needed. Commonly used Au seed layers and nickel (Ni) diffusion barriers can react with the Au-Sn material, shifting the alloy composition; their impact on the stoichiometry must therefore be considered.

#### 3.1.2. Au-Si Eutectic Bonding

**Figure 5:**
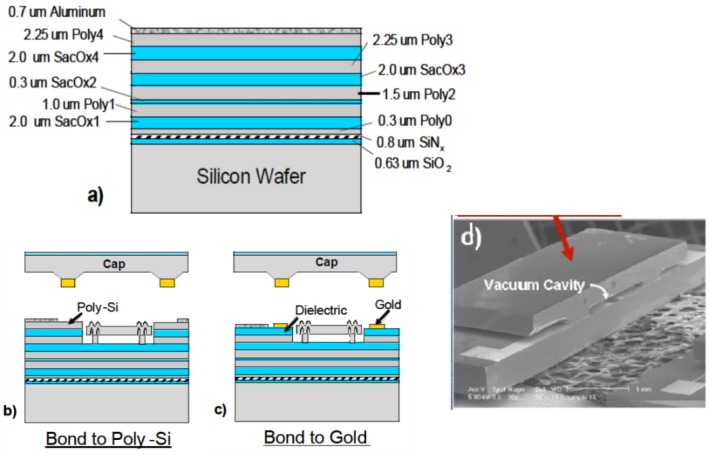
A wafer-level vacuum packaging (WLVP) approach in which Au_0.82_Si_0.18_ eutectic is formed on the cap wafer and bonded to a wafer containing complementary metal-oxide semiconductor (CMOS) elements and a Pirani vacuum sensor. (**a**) SUMMiT V^TM^ thin film CMOS process; (**b**) Bond directly to poly-Si layer on CMOS wafer; (**c**) Bond to Au layer on CMOS wafer; (**d**) Scanning electron microscope (SEM) image of a successfully packaged sensor. [56] Reprinted with permission from Dr. Jay Mitchell.

Au_0.82_Si_0.18_ eutectic bonding, originally developed and adopted as a die attach process [53], has more recently been demonstrated for transfer printing of light emitting diodes (LEDs) [54] and as a wafer bonding method for absolute pressure sensors [55], a MEMS Pirani vacuum gauge with CMOS elements [56,57,58], and other MEMS and smart sensors using surrogate wafers [56,57,58,59,60,61,62,63,64,65,66]. In this approach, Au is deposited by evaporation, sputtering, or electroplating. The source of Si is a deposited Si layer or the Si substrate itself. For smart sensor applications, diffusion barriers are included to prevent Au diffusion into the CMOS device layers. During bonding, the wafers are heated to around 400 °C, and the Au and Si undergo solid-state diffusion until the eutectic composition is reached. Since solid-state diffusion is required, the Au and Si layers are typically formed on the same wafer, ensuring intimate contact between the layers. As shown in Figure 5, once the Au_0.82_Si_0.18_ liquid forms, in this case, on the cap wafer, bonding occurs between the liquid on the cap wafer and the mating Si or Au surfaces on the device wafer. Advantages of this approach include a readily available materials set, ease of deposition, compatibility with MEMS release and cavity etch processes, and the lack of a native oxide on the Au surface.

In addition to the diffusion barrier on the CMOS wafer, an additional diffusion barrier is needed between the Au and Si layers. This barrier prevents premature Si migration into Au, which can occur at temperatures as low as 50 °C [67,68,69]. The premature mixing can cause layers of undesirable SiO_2_ or Si-rich alloys to form on the Au-Sn bonding surface prior to bonding. Early research reports showed the use of titanium (Ti) and/or chromium (Cr) diffusion barriers that were effective in preventing Au-Si solid-state diffusion until temperatures exceeded 500 °C [53]. Only when the temperature exceeds 500 °C, the barrier fails and the desired Au_0.82_Si_0.18_ eutectic forms, enabling the bonding. This temperature is well above CMOS thermal ceilings, so the seal structure would not be compatible with the WLVP of smart sensors. More recently, bonding at 400 °C was demonstrated with the lid metallization stack that included Si; a thin, native SiO_2_ layer; 200 nm of Cr; 500 nm of Au; and microns of plated Au. The combination of SiO_2_ and the Cr diffusion barrier was effective only up to 350 °C for 10 min [56] and was therefore compatible with bonding at temperatures within the CMOS thermal budget. The premature mixing can also be prevented by placing the Au and Si on separate wafers prior to bonding [54,70]. In this embodiment, wafer planarity and parallelism must be well controlled to ensure that all the bonding surfaces across the wafer are in intimate contact for the eutectic to form.

#### 3.1.3. Aluminum-Germanium (Al-Ge) Eutectic Bonding

The Al_0.72_Ge_0.28_ eutectic bonding approach has been demonstrated for 3D ICs [71], LEDs [72], passive test vehicles [73,74,75,76,77,78,79,80], and smart gyroscopes [5,81,82]. Similar to the Au-Si approach, this sealing method relies on solid-state diffusion to form the eutectic solution. Layers of Al and Ge are deposited on one or both wafers, brought into contact with one another, and heated above the eutectic temperature of 423 °C.

**Figure 6:**
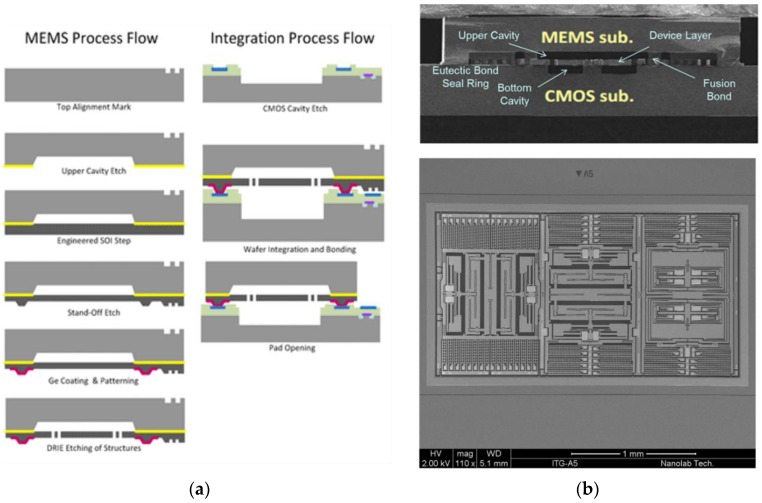
(**a**) Heterogeneous process flow in which germanium (Ge) is deposited on the microelectromechanical (MEMS) wafer and joined to aluminum (Al) bond pads and the seal ring on the complementary metal-oxide semiconductor (CMOS) wafer. The Al_0.72_Ge_0.28_ eutectic bond creates the vacuum package, and the CMOS-to-MEMS interconnects simultaneously. Reprinted with permission from InvenSense; (**b**) Scanning electron microscope (SEM) image of a gyroscope fabricated using the process flow. Reprinted with permission from InvenSense.

To create the desired eutectic material, Al and Ge are deposited at a ratio of roughly 1.0 μm of Al to 0.59 μm of Ge [73]. The deposition methods include evaporation and sputtering, and are followed by typical photoresist or shadow-mask patterning techniques. The native Ge oxide is easily removed with a dilute hydrofluoric (HF) acid solution. The native Al oxide is more difficult to remove effectively. In many cases, Ge is deposited on top of the Al without breaking vacuum to prevent the Al oxide formation. Placing the Al-Ge junction on the same wafer ensures intimate contact between the two metals, facilitating the solid-state diffusion and eutectic formation during heating. To prevent mixing of the sealing materials and Si, SiO_2_ or tantalum nitride (TaN) can be used as diffusion barriers. Figure 6 shows the process flow, in which the MEMS and CMOS elements are fabricated and bonded in the same foundry [81]. In this heterogeneous SoC approach, the wafer-to-wafer bond with the Al_0.72_Ge_0.28_ seal forms the vacuum package and creates interconnects between CMOS and MEMS circuits to create a MEMS gyroscope [82]. The process is now offered by multiple CMOS foundries [83]. The proximity of the bonding temperature to CMOS thermal limits is a primary disadvantage of this approach. Solid-state bonding below the eutectic temperature has been shown to reduce bonding temperatures but at the expense of significant increases in bonding time and force [84].

### 3.2. SLID Bonding

**Figure 7:**
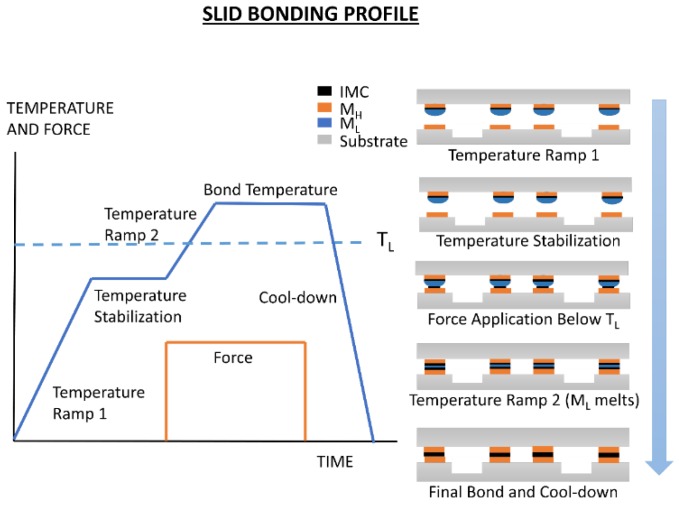
Typical solid-liquid interdiffusion (SLID) bonding process flow. Wafers are heated to a temperature below the bonding temperature, mechanical force is applied, and the wafers are heated above the low melting temperature (T_L_). Naming convention adopted from Hoivik et al. [86].

SLID bonding—also known as transient liquid-phase (TLP) bonding, off-eutectic bonding, and isothermal bonding—involves the use of a metal with a low melting temperature (M_L_) sandwiched between a metal with a higher melting temperature (M_H_), as shown in Figure 7. The melting temperatures of these metals are denoted as T_H_ and T_L_, respectively. When the applied temperature exceeds T_L_, M_L_ melts and wets to M_H_. Although solid-state diffusion occurs below the melting temperature, once M_L_ melts, diffusion rates between M_L_ and M_H_ increase by as much as three orders of magnitude [85]. The solid-liquid diffusion leads to the formation of solid solutions and/or intermetallic compounds (IMCs) made up of M_H_ and M_L_ (since the majority of compounds discussed in this section are intermetallic compounds [IMCs], we will generally refer to all alloys formed in the solid-liquid interdiffusion [SLID] processes as IMCs). The melting temperatures of these IMCs are significantly above T_L_. When bonding is complete, all of the M_L_ should ideally be converted into IMCs, with layers of M_H_ remaining on either side. Excess M_H_ ensures that no reaction occurs between the USM layer and the seal. Excess M_H_ absorbs mechanical stress, and ensures that all of M_L_ can be converted, creating a thermodynamically stable seal [86].

The deposition of pure elemental phases required for SLID bonding is typically much less complex than alloy deposition required for eutectic bonding. In addition, due to the IMC layers high remelting temperature, this approach is well suited for smart sensors that require downstream elevated-temperature processes such as post-bond getter activation and/or assembly. It is also an ideal bonding method for sensor fabrication schemes that require subsequent wafer or chip stacking, as it allows the same bonding temperatures and materials to be used repeatedly. Three different SLID material sets have been successfully demonstrated for the packaging of IC, MEMS, and smart sensing devices. These include copper (Cu)-Sn, Au-Sn, and Au-indium (In).

#### 3.2.1. Cu-Sn SLID Bonding

**Figure 8:**
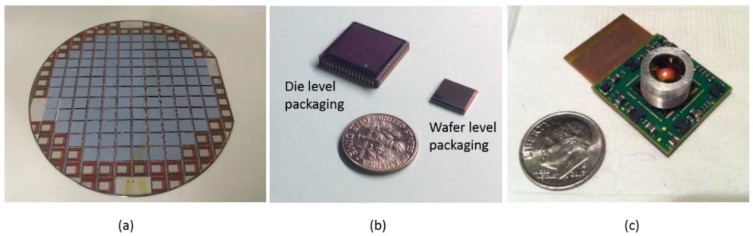
Cu-Sn solid-liquid interdiffusion (SLID) bonding technique used to wafer-level vacuum packaging (WLVP) infrared image sensors. Window wafers with thin film getters and antireflective coatings were bonded to read-out integrated circuit (ROIC) wafers with monolithically integrated microbolometer arrays [103]. (**a**) Photograph of a bonded wafer pair in which window silicon (Si) in the streets between the devices has been removed to allow the devices to be probed at the wafer level; (**b**) Side-by-side comparison of infrared sensors packaged with the die-level and wafer-level packaging approaches; (**c**) Completed WLVP infrared imaging system.

**Figure 9:**
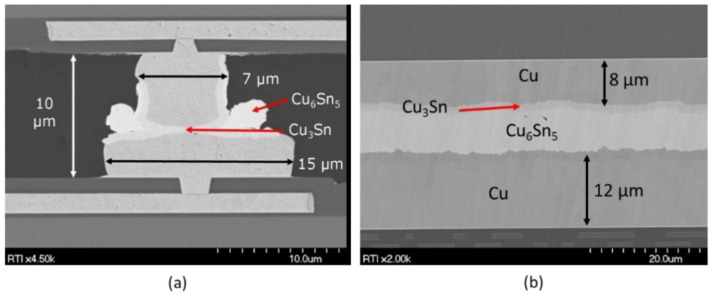
(**a**) Copper-Tin (Cu-Sn) SLID bonding used in integrated circuit (IC) interconnect bonding; (**b**) Cu-solid-liquid-interdiffusion (SLID) bonding used in the wafer-level vacuum packaging (WLVP) of infrared focal plane arrays.

The Cu-Sn material set is the best understood of the SLID sets. This approach has been heavily researched for fine pitch and 3D IC interconnects [86,87,88,89,90,91,92], but has more recently been demonstrated for WLVP MEMS devices [93,94,95,96,97,98,99], smart sensor surrogates [86,100,101,102], and the smart sensor microbolometer devices seen in Figure 8 [103,104]. Figure 9 shows examples of this approach used for both IC interconnects and the WLVP of an infrared imaging sensor.

Cu-Sn SLID bonding involves the use of Cu as M_H_ and Sn as M_L_. Typical bonding temperatures range from 250 °C to 300 °C. USM deposition and material selection are simplified due to the layers of thick Cu that remain following the bonding; these layers prevent mixing between the USM and IMC seal material. Deposition of the elemental Cu and Sn by electroplating is a well-understood, relatively simple, and cost-effective process.

Although native oxides grow on Sn and Cu surfaces, they can be treated without the use of flux. Various dilute acids can be used to remove Cu oxide prior to bonding. The native Sn oxide is thin and can be easily broken apart with a few MPa of mechanical pressure and absorbed by the bond [105]. Forming gas can also be used to treat the Sn oxide [106]. To eliminate the need for any prebonding surface treatments, one can deposit a thin layer of Sn on the bare Cu during the deposition phase, followed by an anneal to form an oxide-resistant IMC layer [107]. Alternatively, a thicker Sn layer can be used to form a symmetric Cu-Sn-to-Cu-Sn structure [108].

**Figure 10:**
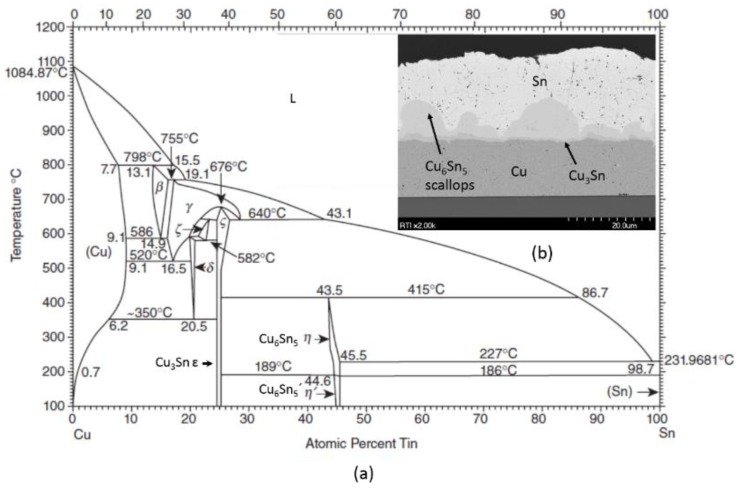
(**a**) Copper-Tin (Cu-Sn) phase diagram—Cu_3_Sn and Cu_6_Sn_5_ intermetallic compounds (IMCs) dominate at the bonding temperatures of interest; (**b**) Scanning electron microscope (SEM) micrograph of a Cu-Sn layer that has been thermally aged at roughly 200 °C for hours. The micrograph depicts both IMC layers and the scalloped nature of the Cu_6_Sn_5_ layer.

As shown in Figure 9 and the phase diagram in Figure 10, two Cu-Sn IMCs, Cu_3_Sn and Cu_6_Sn_5_, form during bonding at the temperatures of interest. The Cu_6_Sn_5_ layer dominates in the early phases of heating. As shown in the scanning electron microscope (SEM) image in Figure 10, the diffusion front of the Cu_6_Sn_5_ IMC tends to be scalloped, which can lead to voiding due to the localized consumption of elemental Sn prior to the application of mechanical force [96]. To prevent voiding, enough elemental Sn must be deposited to account for the growth of scalloped Cu_6_Sn_5_ IMC during any prebond thermal treatments (e.g., outgassing, getter activation, or heat ramp before the force application) [109].

**Figure 11:**
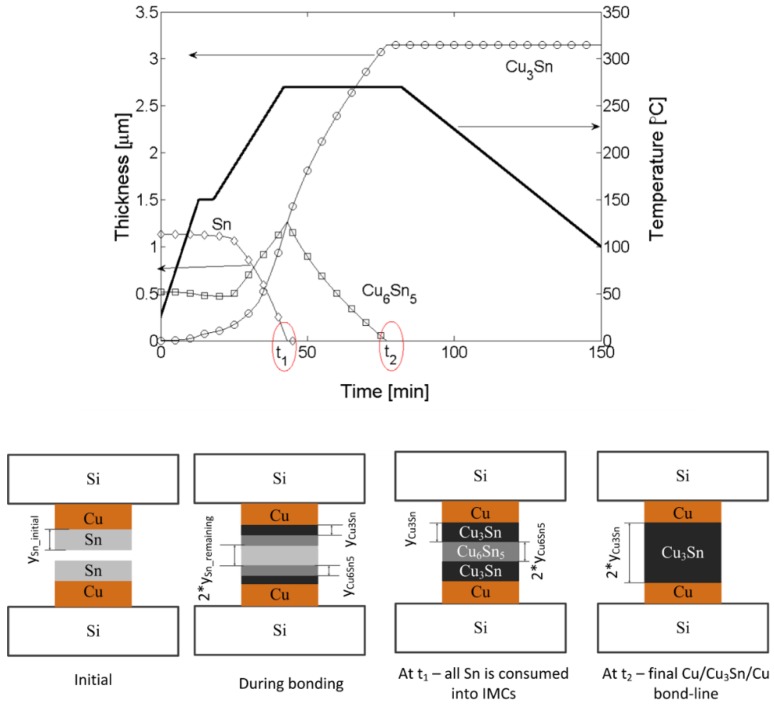
Example output of the copper-tin (Cu-Sn) solid-liquid interdiffusion (SLID) bonding model. Input variables include the temperature profile and thickness of metal layers. In this case, the model predicts that it will take less than 50 min to consume the Sn and roughly 75 min to convert the Cu_6_Sn_5_ into the Cu_3_Sn [110,111,112]. Reprinted with permission from Dr. Thi Thuy Luu.

Although free Sn is needed to prevent voiding in the bond, excess free Sn can cause squeeze-out of Sn outside of the bonding areas. In addition, as the Sn thickness increases, more thermal energy (i.e., longer bond times and/or higher bond temperatures) is needed to form a thermodynamically stable Cu/Cu_3_Sn/Cu seal. Therefore, layer thicknesses, prebond thermal treatments, and bonding profiles must be carefully balanced to generate void-free seals with the desired Cu/Cu_3_Sn/Cu structure. The kinetics of these IMC formations have been studied extensively; the studies produced a model that can be used to optimize layer thicknesses, bonding profiles, and anneal times [110,111,112]. Figure 11 includes an output example from this model.

In addition to the potential void formation from the scalloped shape of the Cu_6_Sn_5_ IMC, voids from other sources are common in the Cu-Sn IMC and interfacial layers. These voids are often ascribed to the Kirkendall effect [113,114].

**Figure 12:**
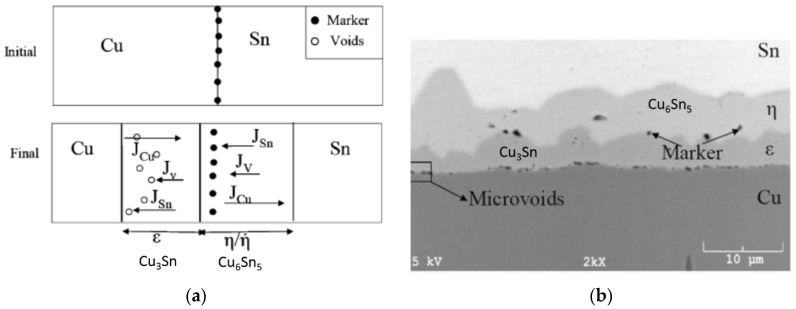
(**a**) Schematic illustrating the Kirkendall void formation in the copper-tin (Cu-Sn) diffusion couple. Relative flux of Cu and Sn interdiffusion can be determined using inert markers. The diffusion rate of Cu into intermetallic compound (IMC) layers exceeds the rate of the diffusion of Sn into IMC layers leading to vacancies at the Cu/IMC interface; (**b**) Corresponding scanning electron microscope (SEM) micrograph depicting marker location and void formation [119]. Reprinted with permission from the Journal of Phase Equilibria and Diffusion.

As shown in Figure 12, Kirkendall voids form due to the imbalance of fluxes *J* of migrating atoms. In the Cu-Sn SLID example, Cu diffuses into the IMC layers at higher rates than Sn, leading to vacancies that coalesce into voids under the right conditions. Recent work has shown that voiding is strongly tied to the Cu deposition method; electroplated Cu films develop significantly more and larger voids than other high-purity films [115,116]. Furthermore, sulfur content in the electroplated deposit has been shown to increase the number of voids and their propensity to coalesce into a continuous layer at the Cu/Cu_3_Sn interface [117,118]. This correlation to the electroplating chemistry may explain the wide range of reported reliability characteristics of the Cu-Sn bonds (results of tests such as drop, electromigration, and contact resistance).

The IMCs formed during the SLID bonding have some well-documented advantages over traditional solder bonding: higher shear strength, tensile strength, electromigration resistance, and creep resistance values [120,121,122,123]. However, as shown in Table 5, Cu_3_Sn and Cu_6_Sn_5_ are stiff (high Young’s modulus) materials. As such, they absorb smaller portions of mechanical stress resulting from the mismatch of the coefficient of thermal expansion (CTE) and/or volumetric shrinkage of the seal material, than common solder materials. (When Cu-Sn bonding is used to create interconnects between IC die, underfilling of the bonding structure with low modulus polymers provides a stress buffering mechanism [124,125].) Taklo et al modeled the effect of mechanical stress in Cu-Sn SLID seals on adjacent CMOS layers and concluded that active devices could be impacted by the seals [101]. It should be noted that the authors used the worst-case values for mechanical properties of Cu-Sn IMCs, hence the results of the model represent a worst-case scenario of the effect of the seals. The authors suggest that thicker layers of the softer elemental Cu could be used to increase the mechanical compliance and reduce the stress imparted on the CMOS layers.

#### 3.2.2. Au-Sn SLID Bonding

Au-Sn SLID has recently been recently studied for the packaging of high-temperature, wide-bandgap semiconductor devices for space and automotive applications [128,129,130,131,132]. Due to the high operating temperatures of these devices, CTE mismatch can create significant stresses in the device and interconnect layers. As shown in Table 6, the high melting temperature and relative low stiffness of the Au-Sn IMCs make Au-Sn SLID suitable for this application. The use of Au-Sn SLID bonding has also been demonstrated for the WLVP of MEMS and smart sensors using passive test vehicles [34,93,94,99,133].

In this approach, Au and Sn act as M_H_ and M_L_, respectively. These layers are most commonly deposited via electroplating or evaporation. Au_0.8_Sn_0.2_ eutectic preforms can also be used in place of Sn. However, for the WLVP of smart sensors, electroplating through photolithographically created templates is preferred due to the need for small feature size and low cost. As in the eutectic Au-Sn approach, the Au layers do not oxidize, enabling fluxless bonding. Unlike in the Au_0.8_Sn_0.2_ eutectic approach, strict composition control is not required.

The primary challenges associated with the Au-Sn SLID bonding include high diffusivities of the Au-Sn couple and the multitude of possible IMC phases. Diffusivities of atoms in the Au-Sn structure have been calculated to be four orders of magnitude larger than their Cu-Sn equivalents [99,119,134]. During thermal treatments (e.g., outgassing, MEMS release, heat ramp to bonding temperature), the high diffusivities cause the relatively thin elemental Sn layers to fully convert to Au-Sn IMC layers prior to bonding. Due to this premature alloying of Sn, the liquid layer is typically made up of Au_0.8_Sn_0.2_ eutectic instead of elemental Sn; therefore, bonding temperatures need to be 280 °C–350 °C to ensure that a liquid layer is formed as the alloy composition passes through the eutectic composition. Several publications describe the kinetics of this process [134,135,136,137].

**Figure 13:**
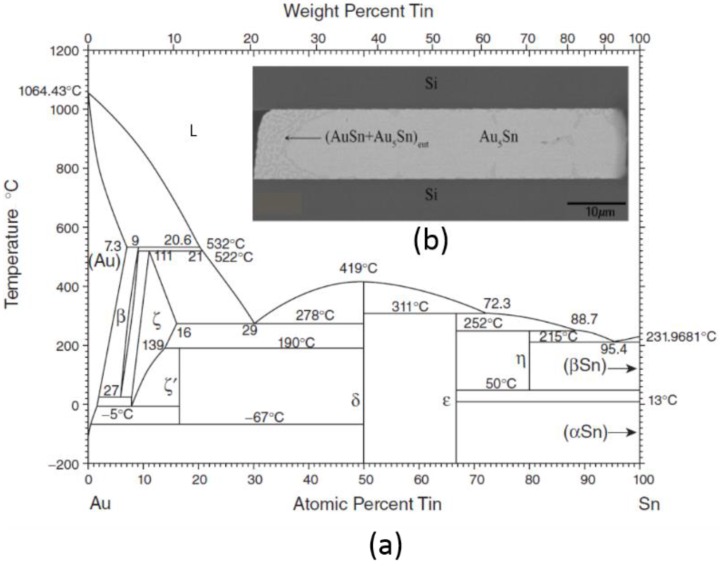
(**a**) Au-Sn phase diagram. Six different intermetallic compound (IMC) layers can form during Au-Sn solid-liquid interdiffusion (SLID) bonding; (**b**) Scanning electron microscope (SEM) image of Au-Sn SLID seal [123]. Reprinted with permission from the Minerals, Metals, and Materials Society.

A comparison of Figure 10 and Figure 13 shows that the Au-Sn phase diagram is more complex than the Cu-Sn phase diagram at the temperatures of interest. In Cu-Sn SLID, only two IMC layers form, whereas six Au-Sn IMC layers are possible in the Au-Sn SLID process. Interconnect failures have been attributed to the formation of the Sn-rich AuSn_4_ IMC [138,139], so the final IMC layers should be made up of more desirable Au-rich IMC layers. The Au-rich seal typically consists of a combination of ζ’ (Au_5_Sn), ζ (nonstoichiometric AuSn), and/or β (Au_10_Sn) IMC layers. The exact seal structure is dependent on a number of factors including initial Au and Sn thicknesses, thermal exposures prior to bonding, and bonding parameters such as temperature, time, and ramp and cooling rates. Due to the large volumes of Au required to form the β phase, the Au-rich IMC layers (i.e., ζ’ [Au_5_Sn] and ζ [nonstoichiometric AuSn]), are more common, but no standard seal structure has been established.

One can use Equation (1) to calculate the ratio of thicknesses of A and B metals required to form an AxB_Y_ IMC:

(1) $$\frac{h_{Α}}{h_{Β}}=\frac{V_{Α}}{V_{Β}}\;=\;\frac{x\;\times \;{Μ}_{Α}\times \;\rho_{Β}}{y\;\times \;{Μ}_{Β}\times \;\rho_{Α}}$$

where h_A_ and h_B_ denote the layer thickness of pure A and B metals, respectively; V_A_ and V_B_ their respective volumes; M_A_ and M_B_ their respective molar masses; and ρ_A_ and ρ_B_ their respective densities. When this formula is applied to a seal consisting of Au_5_Sn sandwiched between elemental Au layers, the Au thickness must be at least three and a half times the Sn thickness. If the β (Au_10_Sn) phase dominates the IMC formation, the Au-to-Sn ratio must be greater than seven. By comparison, Cu_3_Sn is the most Cu-rich IMC at the temperatures of interest. Assuming the final bond consists of Cu/Cu_3_Sn/Cu, a ratio of only 1.3 to 1.0 is required to form this structure. To ensure that alloys on the Sn side of the Au_0.8_Sn_0.2_ eutectic composition are present prior to bonding, and enough collapse occurs to achieve hermetic seals, thicker Sn layers are preferable. However, as Sn thickness increases (assuming Au_5_Sn is the dominate IMC), the Au thickness must increase roughly fourfold with respect to the Sn thickness increase to ensure that elemental Au remains in the bond. This results in cost-prohibitive thicknesses of Au required to generate the preferred Au-AuSn IMC-Au structure. Almost all research reported to date has produced final bond structures with no pure Au remaining in the bond. Since no elemental Au remains, the USM selection issues discussed with the Au_0.8_Sn_0.2_ eutectic process apply here as well. Namely, the USM layers must act as diffusion barriers and be wettable by Au-Sn. Often, these wettable barriers interact with the Au-Sn IMCs, further complicating the final seal structure.

If no unreacted Au remains, the Au-Sn IMCs must be able to absorb stresses that develop in the package. The Au-Sn IMCs are not as stiff as the Cu-Sn equivalents (see Table 5 and Table 6), and are therefore believed to be better stress absorbers. To the authors’ knowledge, no research has been reported on the potential impact on underlying CMOS layers for Au-Sn SLID bonding.

#### 3.2.3. Au-In SLID Bonding

Due to the low melting temperature of In (156 °C), the Au-In SLID bonding approach is attractive for temperature-sensitive devices. This approach has been demonstrated for the packaging of high-temperature, wide-bandgap devices [129,140,141,142], image sensors [143], and in-chip stacking applications [144,145,146]. More recently, the Au-In approach has also been demonstrated for WLVP using passive substrates [147,148].

In this approach, Au and In act as M_H_ and M_L_, respectively. Bonding temperatures range from 165 °C to 200 °C, although solid-state diffusion bonding has been demonstrated at 150 °C [149]. In can be deposited via evaporation, plating, or preforms. However, In oxide is difficult to remove, so the overwhelming majority of Au-In SLID research makes use of an in situ, evaporated Au layer on top of In to prevent oxidation [150]. After deposition, Au and In diffuse, creating an oxide-resistant AuIn_2_ capping layer.

Solid-state diffusion of the Au-In diffusion couple has been shown to occur at temperatures as low as −50 °C with significant diffusion rates at room temperature [151]. This observation is supported by reported activation energies of roughly 0.23 electron volts (eVs), compared to two to three times larger values for Cu-Sn and Au-Sn, respectively [145,151]. To prevent this premature interdiffusion, In and Au layers are commonly deposited on separate substrates. Another approach involves the use of an In-to-Au thickness ratio greater than 3.1 to ensure that some elemental In remains after the interdiffusion is complete [112]. A thin layer of Ti has also been shown to prevent interdiffusion of the Au and In at low temperatures and allow interdiffusion at the bonding temperatures [145].

**Figure 14:**
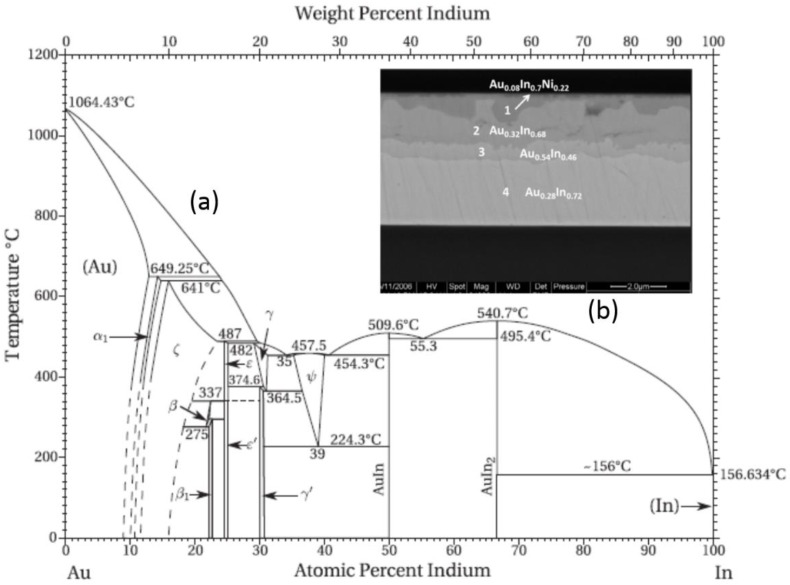
(**a**) Gold-Indium (Au-In) phase diagram; (**b**) Scanning electron microscope (SEM) micrograph depicting a seal structure with four different Au-In intermetallic compound (IMC) phases. Au-In-Nickel (Ni) IMC is shown in phase 1, likely the γ′ Au-In in phase 2, likely AuIn in phase 3, and AuIn_2_ in phase 4 [148]. Reprinted with permission from the Institute of Electrical and Electronics Engineers (IEEE).

The Au-In phase diagram shown in Figure 14 is complex; seven different IMC phases exist at the temperatures of interest. When Equation (1) is applied to this alloy, Au-to-In thickness ratios ranging from 1.5 to 7.1 are required to form Au-rich IMC layers (e.g., γ′, ɛ, β_1_, ζ, and α_1_). Similar to the Au-Sn approach, these Au-rich IMCs, combined with relatively thick In layers, result in a seal structure that lacks elemental Au. Although no standard process has been developed, the majority of research has resulted in a final seal consisting of AuIn and AuIn_2_ layers. Initial research suggests that these are relatively soft, elastic, low-modulus IMCs [152,153]. No elemental Au remains, so the adjacent USM materials must be compatible with the Au-In IMCs.

### 3.3. Metal-to-Metal Thermocompression Bonding

**Figure 15:**
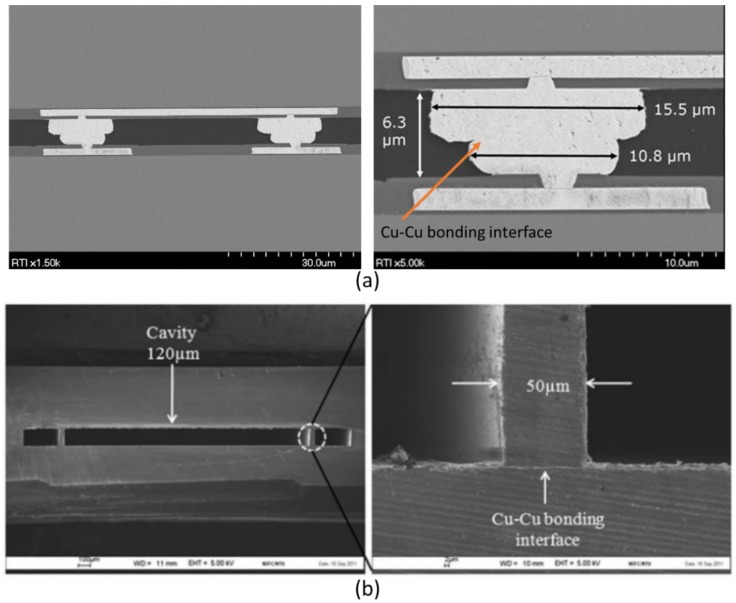
(**a**) Scanning electron microscope (SEM) micrographs of copper (Cu)-Cu bonded interconnect; (**b**) SEM micrographs of Cu-Cu bonded hermetic seal [160]. Figure 15b reprinted with permission from Microsystem Technologies.

Metal-to-metal bonding, also known as diffusion bonding, relies on solid-state diffusion between metals at elevated temperature and pressure. Typically, the same metal is used on each wafer, and a homogenous metal seal is formed by self-diffusion (e.g., Al-Al, Cu-Cu, and Au-Au). Like the other proposed sealing methods, the metal-to-metal approach has also been researched for IC interconnects [92,154] and WLVP on proxy surrogates [155], MEMS accelerometers [156], and infrared imagers [157]. Embodiments that simultaneously create IC interconnects and hermetic seals have also been demonstratedrff on surrogate wafers [158,159,160,161,162]. Figure 15 depicts examples of metal-to-metal bonds used for both interconnects and hermetic seals.

Compared to the eutectic and SLID sealing methods that involve phase transformations and produce various alloys, this approach is less complex and more flexible with respect to upstream and downstream WLVP processes. No liquid layer forms during bonding, so wetting is not a concern, simplifying USM material selection. Deposition of the seal material is also relatively straightforward and typically employs evaporation, electroplating, and/or sputtering techniques.

Typical bonding variables include mechanical pressure, and temperature ramps and dwells. Mechanical scrubbing and/or ultrasonic vibration are sometimes used to assist the self-diffusion. Unlike the eutectic and SLID approaches, which rely on interdiffusion, the metal-to-metal seal material is not typically impacted by prebond thermal treatments (e.g., MEMS release and outgas bakes). The lack of interdiffusion also eliminates the risk of Kirkendall voiding.

Due to the lack of a liquid layer, this approach is more sensitive to nonplanarity and oxide formation. To ensure that the sealing surfaces across the entire wafer are in contact, a higher degree of planarity is required. Chemical mechanical planarization (CMP) and/or uniform deposition methods (e.g., sputtering and evaporation) are often used in conjunction with thermocompression bonding to ensure uniform contact of the sealing surfaces. In addition, soft metals with relatively low yield strengths and low melting temperatures should be chosen. This allows the metals to plastically deform at CMOS- and MEMS-compatible temperatures and pressures. This deformation can compensate for small amounts of nonplanarity between the sealing surfaces. The metals most commonly evaluated for this approach are Al-Al, Cu-Cu, and Au-Au.

In addition to planarity requirements, another concern for metal-to-metal sealing of smart sensors is the presence of the native oxide. In the eutectic and SLID approaches, the native oxides can be broken up and absorbed by the collapse of an underlying liquid layer. However, in thermocompression bonding, the oxides must be more thoroughly removed prior to bonding. One of the primary advantages of the Au-Au solution is the lack of a native oxide. Although Cu does have a native oxide, it is easily removed in dilute acids applied in situ or ex situ of the bonding process. Self-assembled monolayer (SAM) technology that prevents oxide growth has also been demonstrated for Cu-Cu thermocompression bonding [161,163,164,165]. Al is a soft metal commonly used as a contact pad material for CMOS processing and is therefore an attractive choice as well. However, the native Al oxide is difficult to remove, requiring higher bonding temperatures and pressures [156]. In addition, Al’s relatively high CTE can lead to more stress in Si packages due to higher levels of the CTE mismatch.

Bonding temperatures are a function of the metal selection, pressure, planarity, and surface cleanliness. Bonding pressures are usually between 0.2 and 10.0 MPa with bonding times varying from 30 to 120 min. Postbond anneals are often used to continue the solid-state diffusion process, ensuring a hermetic, mechanically strong seal. As discussed in previous sections, the added bonding and anneal time may increase gas pressure inside the package and must be balanced accordingly. Metals with relatively high self-diffusivities and low activation energies should be chosen to minimize the bonding time and temperature. Table 7 lists properties of the seal materials and the typical bonding temperatures.

## 4. Thin Film Getters

Over time, the pressure inside the vacuum package will rise due to a combination of leakage through the enclosure materials, permeation, and outgassing of the inner surfaces. Due to the small cavity volumes, small changes in gas volume cause large changes in package pressure. MIL-STD-750E and MIL-STD-883H standards define hermetic packages as those with leak rates less than 1 × 10^−6^ to 5 × 10^−10^ mL·atm/s [168,169].

**Figure 16:**
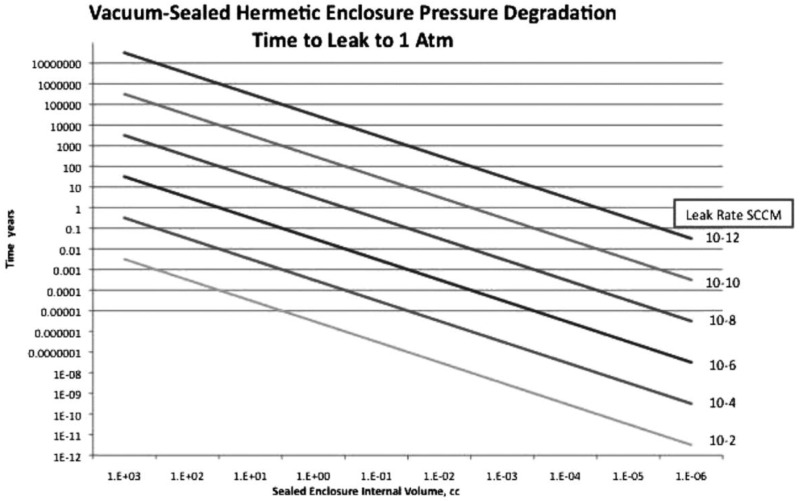
Relationship between the package volume, the leak rate, and the time for the pressure to rise to 1 atmosphere (atm). Small package volumes used in state-of-the-art sensors leak up to atmospheric pressure in seconds to minutes [17]. Reprinted with permission from the Society of Photo-optical Instrumentation Engineers (SPIE).

When these leak rates are applied to modern smart sensors, which typically have package volumes in the nanoliter (1 × 10^−6^ mL) range, the packages could leak up to the atmosphere in as little as minutes (Figure 16). Getters offset the impact of these gas sources.

Getters rely on two mechanisms for sorption, namely chemisorption and physisorption. The majority of gas species (carbon monoxide [CO], carbon dioxide [CO_2_], oxygen [O_2_], nitrogen [N_2_]) react via dissociative chemisorption that leads to the formation of an oxide, carbide, or nitride. When the getter temperature is high enough, the resulting compounds diffuse into the getter bulk, leaving behind a fresh getter surface. This cycle continues until the getter is saturated with the species [17]. In contrast to the gases listed above, hydrogen (H_2_) is physisorbed—due to its low disassociation energy and high rate of diffusion—through the metal lattice. The physisorbed H_2_ forms a solid solution with the getter lattice, characterized by a specific equilibrium pressure. Depending on the H_2_ concentration and ambient temperature, H_2_ can move in or out of the getter lattice.

**Figure 17:**
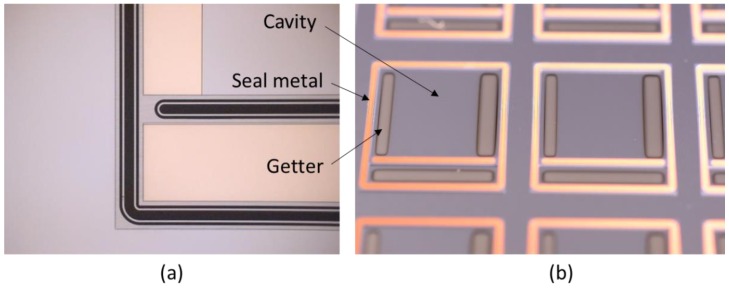
Images of window wafers fabricated for the wafer-level vacuum packaging (WLVP) of infrared imagers. The window wafers were coated with a thin film getter that was patterned using shadow masks during the deposition [103]. (**a**) Optical micrograph showing the location a titanium (Ti)-based thin film getter; (**b**) Optical image depicting a zirconium (Zr)-based getter.

Thin film getters are typically deposited through evaporation or sputtering. As shown in Figure 17, in the case of smart sensors, shadow masks are commonly used to pattern the material. The microstructure of the getter is a key parameter that determines its gas sorption capacity. Increased surface area leads to increased pumping speed; therefore, porous materials are usually preferred. However, porosity must not result in mechanical fragility as semiconductor sensors do not typically tolerate particles. Grain size is another important parameter. Many of chemisorbed gases diffuse along grain boundaries, so smaller grains are often preferred. Deposition conditions (e.g., pressure, temperature, and power) can be used to tailor the microstructure of the thin film getter. Getters may be fabricated with an oxide, carbide, and/or nitride capping layer to passivate the getter until activation. During activation, the getter is heated, and the passivation layer diffuses into the bulk, beginning the sorption cycle described above.

Getter activation temperatures for smart sensors should remain below 450 °C for CMOS compatibility. Commercially available solutions applicable to smart sensors are offered by both SAES Getters and NanoGetters, with activation temperatures between 300 °C and 400 °C. In most cases, the getter material is activated during the sealing process. Getter reactivation after sealing is commonly implemented for additional capacity. These reactivation processes typically consist of relatively low-temperature (150 °C–300 °C), long-time (hours) anneals.

If the smart sensor contains components incompatible with getter activation temperatures, a separate getter activation step can be completed prior to sealing. In this case, the getter is deposited on the lid wafer, which does not contain the temperature-sensitive components. The lid wafer is then independently heated to the activation temperature and subsequently cooled to the bonding temperature. The getter pumps gases in the vacuum chamber between activation and sealing, so the chamber pressure should be minimized to prevent premature saturation of the getter surface prior to sealing. Getters with low activation temperatures (e.g., as low as 200 °C) have been developed for temperature-sensitive applications [170].

Getter operation is often quantified by the pumping speed and the sorption capacity for various gas species. As mentioned previously, the capacity is a function of the getter stoichiometry and volume. The pumping speed is limited by the rate at which the gas molecules stick to the surface and/or diffuse into the bulk. In the typical scheme, in which sealing and activation occur in parallel, getters should have high affinities and diffusivities for the gases of interest. If activation occurs prior to bonding, getter materials with lower sticking probabilities may be desired to prevent premature surface saturation by gases in the vacuum bonding chamber.

**Figure 18:**
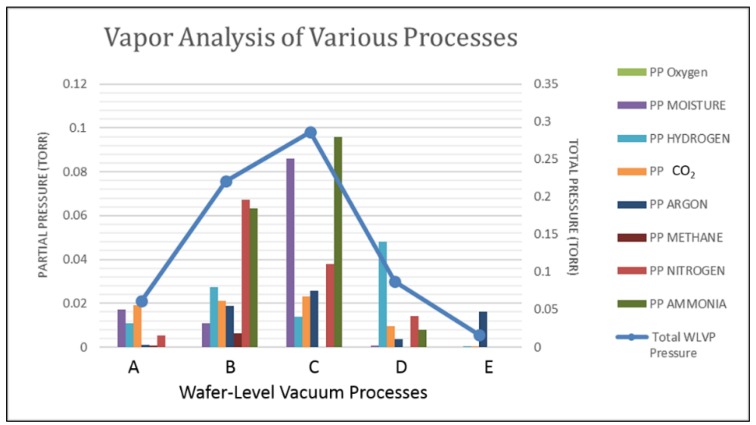
Spectra data from a high-resolution internal vapor analysis (HR-IVA) that was converted into partial and total package pressure values [103].

Since getter pumping speeds and capacities are a function of the various diffusivities and sticking coefficients, it is important to understand what gas species are present in the package. In addition, some gases will not react with the getter and must be removed through other means. Residual gas analysis (RGA) of the bonding chamber, and testing of outgassing from bulk materials can provide some insight into what gases are present in the package. To quantify the gas constituents inside the small smart sensor packages, Oneida Research Services (ORS, Whitesboro, NY, USA) has developed a high-resolution internal vapor analysis (HR-IVA) capable of analyzing package volumes as small as 1 × 10^−6^ cc [171]. The test involves puncturing the package in a vacuum chamber. The released gas is analyzed by a time-of-flight mass spectrometer. As shown in Figure 18, in addition to the data on the concentrations of various species, one can estimate the total pressure in the package if enough gas is released during the puncture process [172]. Once the gas constituents are known, getter materials and outgassing processes can be optimized.

## 5. Outlook and Conclusions

The need to reduce sensor size, weight, power, and cost (SWaP-C) is causing a shift in packaging architectures from multichip approaches, where IC and MEMS are integrated at the board or package level to SoC approaches in which MEMS and IC are integrated much earlier in the fabrication process. In parallel, pixelated sensors, which, due to their need for higher interconnect densities, have long been packaged in SoC architectures, are transitioning from die-level packaging to wafer-level packaging approaches to reduce SWaP-C. These trends are converging on the need for IC-compatible, wafer-level packaging solutions that provide hermetic or vacuum enclosures for SoC smart sensing components. The incumbent WLVP approaches used to package discrete MEMS devices such as anodic, direct, or glass frit bonding are not compatible with the thermal budget of the IC circuitry and/or require too much valuable smart sensor real estate. Device and process designers have been therefore modifying alternative, CMOS-compatible interconnect solutions developed originally for IC integration for the WLVP of smart sensors.

The relative maturity of the CMOS-compatible, metal-based WLVP solution is typically congruent with the relative maturity of the IC solution from which it originated. Of the various bonding techniques discussed, the Cu-Sn SLID, Au-Sn eutectic, and Au-Au and Cu-Cu thermocompression are the most thoroughly researched. Interest in the other bonding approaches is primarily driven by unique device and business model requirements. For example, the Au-In SLID bonding development is aimed at devices with thermal ceilings below 200 °C; the Au-Sn SLID research is motivated by devices with very high operating temperatures, and the Al-Ge eutectic process is a result of fabless business models. Table 8 provides a summary of relative strengths and weaknesses of bonding approaches reviewed in this paper.

There are other traditional and emerging IC packaging techniques that could provide WLVP solutions. Bonding with the ductile and well-understood Pb_0.38_Sn_0.62_ solder could be evaluated for space and military applications that provide Restriction of Hazardous Substances exceptions. Sn_0.97_Ag_0.03_ eutectic, a commonly used lead (Pb)-free solder, may offer another potential WLVP approach. Plasma-assisted dry soldering (PADS), forming gas, and formic acid etching provide well-established techniques to remove native oxides on these materials prior to bonding. Another alternative bonding approach uses nanostructured materials that can be tailored to produce desirable traits such as reduced bonding temperatures and reduced stiffness [173].

Most of the reported research in WLVP has been performed on proxy or surrogate wafers that did not contain functional sensors. Although these studies are useful for demonstrating and narrowing down potential WLVP solutions, the community would benefit from reports of the performance of functional smart sensors packaged using wafer-level techniques. The presence of active devices affects the WLVP outcome through at least two factors. One is the outgassing from the device structure. The second is the potential sensitivity of the device operation to mechanical strains that may be introduced by the presence of the microchambers bonded to the device wafer. The mechanical impact of the package on the sensing device is largely unknown. Many of the equivalent IC interconnect solutions mitigate package-induced stresses through the use of underfills and stress-absorbing polymers, solutions that are not directly applicable to WLVP. A more extensive modeling of thermomechanical behavior of smart sensors in WLVPs is needed to illuminate the effect of stress and potential solutions. The community also needs to gain more insight into the related issues of reliability of packaged components for which reported data are scarce.

The development of a successful WLVP process depends on the capabilities of bonding equipment and getter materials. The majority of commercially available wafer bonding equipment and thin film getter materials were developed for traditional MEMS bonding techniques, and are not as easily integrated into SoC packages. The SoC smart sensors are often more temperature-sensitive and more sensitive to outgassing because of their smaller package volumes. They also provide less surface area for getter deposition and therefore require better patterning techniques to take advantage of all the available area, without contaminating the seals. Complete activation of thin film getters can only be achieved above 400 °C, and only the state-of-the-art bonding systems can provide adequate control of the wafer spacing and temperature. The development of improved patterning techniques for thin-film getters and the development of getters with lower activation temperatures will broadly enable the WLVP of smart sensors. In parallel, WLVP technology will benefit from further proliferation of bonding systems that allow independent heating and cooling of wafers to be bonded, in situ alignment, and lower base pressures. Once mature, these CMOS compatible wafer-level packages could replace the multitude of custom packages that exist currently in the sensor industry (the “one product, one package” phenomenon). The existence of a more standard approach would reduce the cost and the development cycle for smart sensors and enable them meet the challenge and the opportunity brought about by the Internet of Things.

## Figures and Tables

**Table 1:** Comparison of traditional wafer bonding approaches used for discrete microelectromechanical systems (MEMS) with metal bonding approaches more suitable for wafer-level vacuum packaging (WLVP) of smart sensors.

Comparison of Bonding Approaches for Smart Sensors							
	Bonding Approach	Bonding Temperature (°C)	Topography Tolerance	Outgassing	Substrate Flexibility	Seal Area	CMOS Compatibility
**Traditional Approaches**	Anodic	300–450	Low	High	Low	Low	Poor
	Direct	>800	Very low	Mid	Low	Low	Poor
	Glass frit	430–450	High	High	Low	High	Good
**Metal Bonding Approaches**	Eutectic	>200	High	Low	High	Low	Good
	SLID	>200	High	Low	High	Low	Good
	Metal-to-metal	>250	Low	Low	High	Low	Good

**Table 2:** Advantages and disadvantages of the various, metal-based, wafer-level vacuum packaging (WLVP) approaches.

Comparison of Metal-Based Bonding Approaches		
Bonding Approach	Main Advantages	Main Disadvantages
**Eutectic alloy**	Ductile seal	Complex deposition Bonding temperature dependent on alloy stoichiometry Under-seal metal (USM) can react with eutectic alloy
**SLID**	Mature deposition and bonding processes Re-melting temperature is greater than bonding temperature	Lack of ductility in seal
**Metal-to-metal**	Mature deposition and bonding processes Relatively simple deposition and metallurgy	No collapse layer to absorb topography Oxide removal more critical Generally requires higher force and temperature during bonding

**Table 3:** List of the most commonly used and applicable eutectic alloys.

Eutectic Bonding Alloys and Corresponding Eutectic Temperatures			
Alloy	Eutectic Temperature (°C)	Advantages	Disadvantages
**Au_0.8_Sn_0.2_**	280	Relative maturity Fluxless bonding Deposition options	Steep eutectic liquidus lines USM integration challenging
**Au_0.82_Si_0.18_**	363	Ease of deposition	Long bonding times USM integration challenging
**Al_0.72_Ge_0.28_**	423	Seal materials compatible with legacy CMOS processes	Bonding temperatures at or near CMOS limits Difficult to remove native oxide

**Table 4:** Comparison of various Au_0.8_Sn_0.2_ deposition methods.

Comparison of Gold-Tin (Au-Sn) Eutectic Deposition Methods		
Deposition Method	Main Advantages	Main Disadvantages
**Ball laser jetting**	Composition control	Difficult to precisely control seal geometry Serial deposition method
**Electroplating Au_0.8_Sn_0.2_ alloy**	Dimensional control	Poor compositional control Poor bath stability
**Alternating layers of Au and Sn**	Dimensional control	Layer thickness must be controlled to achieve composition target

**Table 5:** Materials properties of copper (Cu)–solid-liquid interdiffusion (SLID) compounds compared to solders commonly used in the integrated circuit (IC) industry [86,126,127].

Materials Properties of Copper-Tin (Cu-Sn) Solid-Liquid Interdiffusion (SLID) Compounds and Common Solders						
Metallurgy	Bonding Temperature (°C)	Compound	Elastic Modulus (GPa)	Yield Strength (MPa)	CTE (ppm K^−1^)	Melting Temperature (°C)
**Cu-Sn SLID**	>230	Cu	110	180	17	1084
		Sn	41	35	23	232
		Cu_3_Sn(ɛ phase)	79–153	1787	19	676
		Cu_6_Sn_5_(ɲ phase)	84–119	2009	16	415
**Common Solders**	>185	Pb0.38Sn0.62 wt%	15.7	30.2	18.7	183
	>220	SnAg0.03 wt%	26.2	50	20	221

**Table 6:** Properties of gold-tin (Au-Sn) solid-liquid interdiffusion (SLID) bonding compounds [86,126].

Materials Properties of Compounds Formed During Slid Bonding					
Metallurgy	Bonding Temperature (°C)	Compound	Elastic Modulus (GPa)	CTE (ppm K^−1^)	Melting Temperature (°C)
**Gold-Tin (Au-Sn) Solid-Liquid Interdiffusion (SLID)**	>280	Au	77.2	14.4	1064
		Sn	41	23	232
		AuSn (δ phase)	70–101	14	419
		Eutectic AuSn (mixture of δ and ζ’)	69–74	16	278
		Au_5_Sn (ζ’ phase)	62–76	18	190
		AuSn_0.18–0.10_ at % (ζ phase)	58	20	519
		Au_10_Sn (β phase)	88	N/A	532

**Table 7:** Properties of metals used in metal thermocompression bonding [166,167].

Properties of Commonly Used Materials for Metal-to-Metal Thermocompression Bonding					
Metal	Bonding Temperature (°C)	Self-Diffusivity (m^2^/s)	Activation Energy (eV)	CTE (μm/m·K at 25 °C)	Melting Temperature (°C)
Aluminum (Al)	>400	4.2 × 10^−19^ (at 500 °C)	1.49	23.1	660
Copper (Cu)	>350	4.2 × 10^−14^ (at 500 °C)	2.19	16.5	1084
Gold (Au)	>260	1.0 × 10^−18^–1.0 × 10^−19^ (at 400 °C)	1.81	14.2	1064

**Table 8:** Summary of the metal-based bonding solutions discussed in this review.

Summary of Bonding Approaches Reviewed				
Bonding Type	Material	Bonding Temperature (°C)	Primary Advantages	Primary Disadvantages
Eutectic	Au_0.8_Sn_0.2_	>280	Relative maturity Fluxless bonding Deposition options	Steep eutectic liquidus lines Challenging under-seal metal (USM) integration
	Au_0.82_Si_0.18_	>363	Ease of deposition	Complex diffusion barrier Challenging USM integration
	Al_0.72_Ge_0.28_	>423	Compatibility of seal materials with legacy complementary metal-oxide semiconductor (CMOS) processes Production process available in CMOS foundries	Bonding temperatures at or near CMOS limits Difficulty in removing native oxide
Solid-liquid diffusion (SLID)	Cu-Sn	>232	Relative maturity	Stiffness of final bonding materials
	Au-Sn	>280	Low modulus bonding materials and high remelting temperature (good candidate for devices with high operating temperatures)	Complex phase diagram USM integration challenging due to high rate of Au consumption
	Au-In	>156	Low bonding temperature	Complex phase diagram Premature mixing of bond materials
Metal-to-Metal	Al	>400	Standard CMOS pad material	Difficulty in removing native oxide High bonding temperature Sensitivity to particles and topography
	Cu	>350	Relative maturity Simplicity	Sensitivity to particles and topography
	Au	>260	No native oxide Relative maturity Simplicity	Sensitivity to particles and topography

## Abbreviations

The following abbreviations are used in this manuscript:

2D: two-dimensional
3D: three-dimensional
Al: aluminum
Au: gold
CMOS: complementary metal-oxide semiconductor (CMOS)
CMP: chemical mechanical planarization
CO: carbon monoxide
CO_2_: carbon dioxide
Cr: chromium
CTE: coefficient of thermal expansion
Cu: copper
eV: electron volt
Ge: germanium
H_2_: hydrogen
HF: hydrofluoric
HR-IVA: high-resolution internal vapor analysis
IC: integrated circuit
IMC: intermetallic compound
In: indium
IoT: Internet-of-Things
MEMS: microelectromechanical systems
MOEMS: micro-optoelectromechanical systems
MPa: Megapascal
N_2_: nitrogen
Ni: nickel
nm: nanometers
O_2_: oxygen
ORS: Oneida Research Services
PADS: plasma-assisted dry soldering
Pb: lead
RF: radiofrequency
RGA: residual gas analysis
ROIC: readout integrated circuit
SAM: self-assembled monolayer
Si: silicon
SLID: solid-liquid interdiffusion
Sn: tin
SoC: system-on-chip
SPIE: Society of Photo-optical Instrumentation Engineers
SWaP-C: size, weight, power, and cost
TaN: tantalum nitride
Ti: titanium
TLP: transient liquid phase
USM: under-seal metal
WLP: wafer-level packaging
WLVP: wafer-level vacuum packaging
Zr: zirconium
